# Proximal humerus reconstruction in orthopedic oncology

**DOI:** 10.20517/2394-4722.2020.94

**Published:** 2021-02-03

**Authors:** Sridhar Pinnamaneni, Timothy A. Damron

**Affiliations:** 1 Shoulder and Elbow Section, Signature Orthopedics, St. Louis, Missouri 63128, USA.; 2 Orthopedic Oncology Section, Upstate Orthopedics, Syracuse, New York 13210, USA.

**Keywords:** Proximal humerus, oncology, primary bone tumor, metastatic disease, endoprosthesis, allograft

## Abstract

Proximal humeral reconstructive options following radical resection of proximal humeral primary and metastatic bone malignancies have evolved over time. With the relatively recent advent of the reverse total shoulder (RTSA), this technique has been increasingly employed in this setting over hemiarthroplasty techniques. An array of options, including proximal humeral allograft-prosthetic composites (including both RTSA and hemiarthroplasty), megaprostheses, and osteoarticular allografts, is reviewed from the perspective of their indications, techniques, complications, and published results. An extensive case-based pictorial presentation illustrates these options.

## INTRODUCTION

The proximal humerus is a common site for metastatic and primary bone tumors^[1,2]^. With the evolution of chemotherapeutics, radiotherapy, and advanced imaging techniques, limb salvage surgery has become the norm in this location except for very advanced cases. Tumors in this region impose significant challenges for local control, reconstruction, and function. Potential glenohumeral joint involvement and close proximity of neurovascular structures affects local control and function, while lack of intrinsic stability and dependence on dynamic and static stabilizers affects reconstruction and function. Resection for metastatic disease may require bone-only resection, but restoration of soft-tissue attachments and glenohumeral stability are challenging. Resection for primary bone tumors may require complete or partial resection of the deltoid, rotator cuff, joint capsule, axillary nerve, and portions of the scapula to achieve oncologic margins. Reconstruction after the latter resection creates even more complex reconstructive issues. The purpose of our article is to provide a comprehensive review of current reconstruction options after proximal humeral resection for oncologic reasons. This review is supplemented with case-based examples.

## CLASSIFICATION AND OUTCOME ASSESSMENT

Classification of shoulder girdle resections according to Malawer range from types I to VI^[3]^. This classification is based on surgical margins, relationship of the tumor to other anatomic compartments, status of the glenohumeral joint, magnitude of the surgical procedures, and status of the abductor mechanism (deltoid/rotator cuff) [Table 1]. Functional and quality of life outcomes after treatment of musculoskeletal tumors are most commonly assessed with the Musculoskeletal Tumor Society (MSTS) score^[4]^. This scale is based on questions about functional outcomes, pain, and emotional status [Table 2]. While it has been shown to be a reliable tool for the upper extremity, the MSTS score can overestimate function as compared to the patient-perceived score^[4]^. As for other sites, complications following these procedures are classified as described by Henderson *et al.*^[5]^ [Table 3].

## PREOPERATIVE CONSIDERATIONS

In general, the orthopedic oncology patient has several specific characteristics that affect the choice of surgical reconstruction. The potential for a shortened survival, particularly in patients with diffuse metastatic disease, myeloma, and some primary bone tumors, should be considered. Orthopedic oncology patients often need a surgical solution that allows for immediate stability to allow restoration of function, especially in patients with poorer prognosis. Concomitant effects of radiotherapy and chemotherapy, including increased risk for scarring, wound healing problems, and infection, should also be considered. These factors differ from a non-oncology orthopedic patient and should be considered prior to finalizing a surgical plan.

The glenohumeral joint significantly relies on the dynamic stabilizers and force-couplers to allow for range of motion of the shoulder, including the rotator cuff, glenohumeral ligaments, and deltoid^[6]^. These structures comprise the functional anatomic compartment of the shoulder^[7]^. During wide resection of the proximal humerus for oncology surgery, major portions of the functional anatomic compartment are often removed. Those portions can specifically include all or the deep portion of the deltoid, subscapularis muscle belly, superior-posterior rotator cuff musculature, latissimus dorsi, brachialis, and portions of the triceps. Wide resection of this functional compartment during surgery creates a complex situation during reconstruction, especially when trying to restore both stability and function to the shoulder and proximal humerus^[7]^. When the rotator cuff is going to be compromised or resected, a reverse total shoulder arthroplasty (RTSA) implant should be considered. Need for resection of the brachial artery and/or all of the three major nerves affecting distal function (radial, median, and ulnar) are considered contraindications for limb salvage surgery, but resection of the axillary nerve is not.

## INDICATIONS

The proximal humerus is a common location for upper extremity tumors. Common tumors requiring resection affecting the proximal humerus include metastatic bone disease, myeloma, primary bone sarcomas, and locally aggressive benign tumors.

### Metastatic Bone Disease/Myeloma

**Figure 1.**
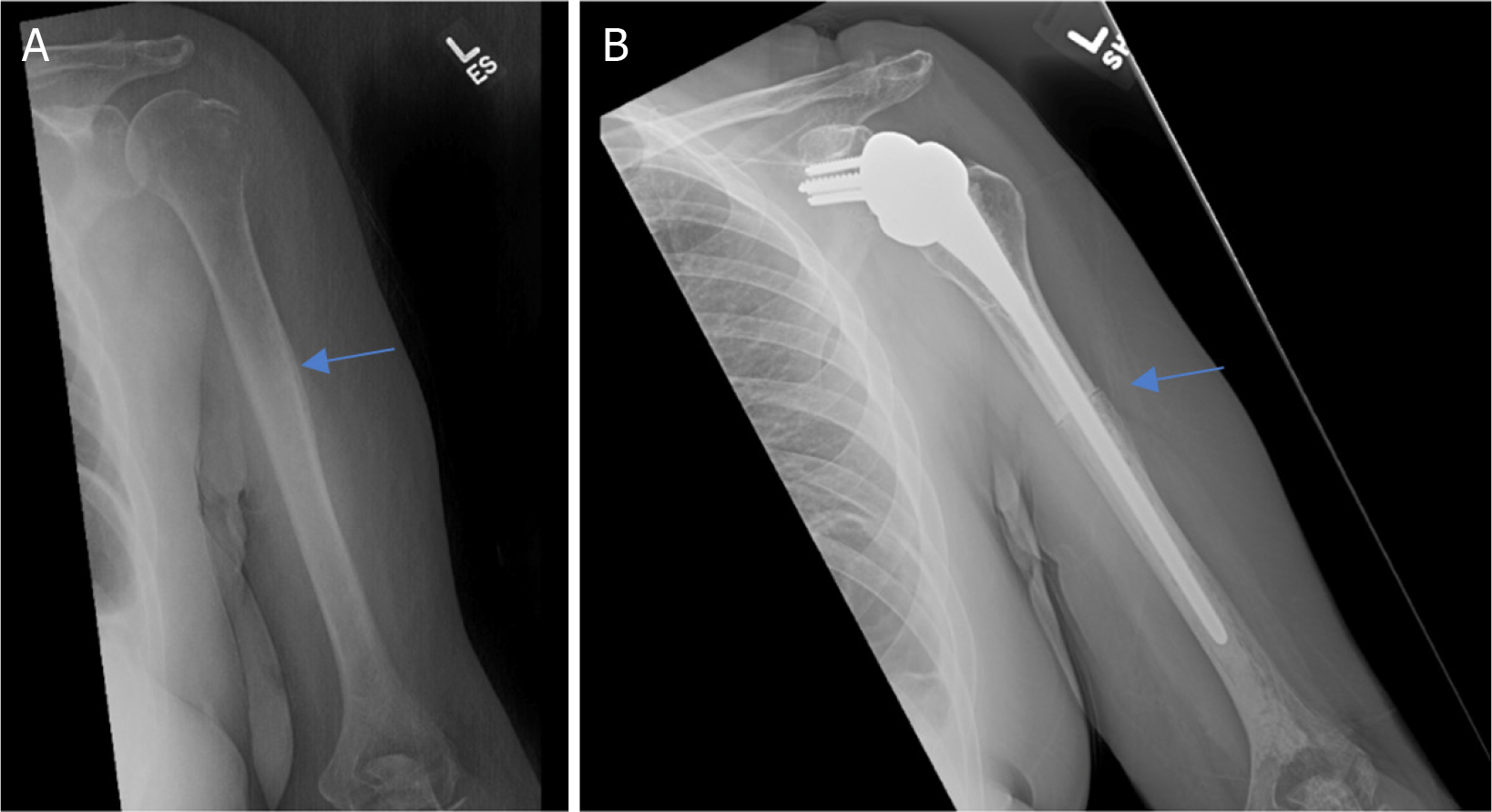
Anteroposterior radiographs of a 50-year-old female with: a solitary renal cell carcinoma tumor of the proximal humerus preoperatively (A); one year after wide excision and proximal humeral APC reconstruction (B). Blue arrow notes the junction between the Allograft and the native bone

**Figure 2.**
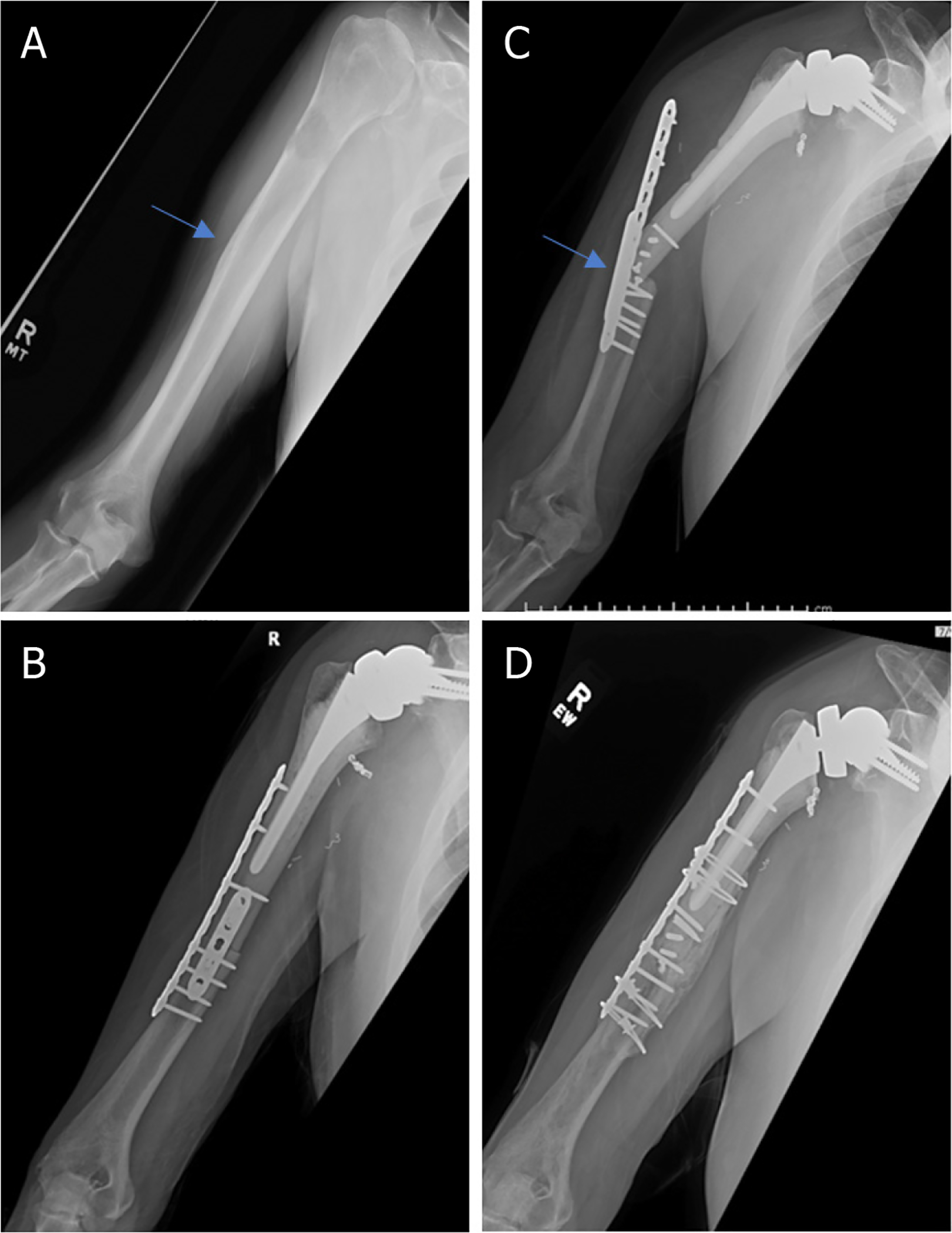
Anteroposterior radiographs of a 76-year-old male with: a solitary renal cell carcinoma tumor of the proximal humerus preoperatively (A); six months after wide excision and proximal humeral allograft prosthetic composite (APC) reconstruction with RTSA (B); one year postoperatively, patient represented with allograft-host junctional nonunion with hardware failure (C); anteroposterior radiographs after bone grafting, repair of the non-union with open reduction, and internal fixation (D) Blue arrow notes the junction between the Allograft and the native bone.

**Figure 3.**
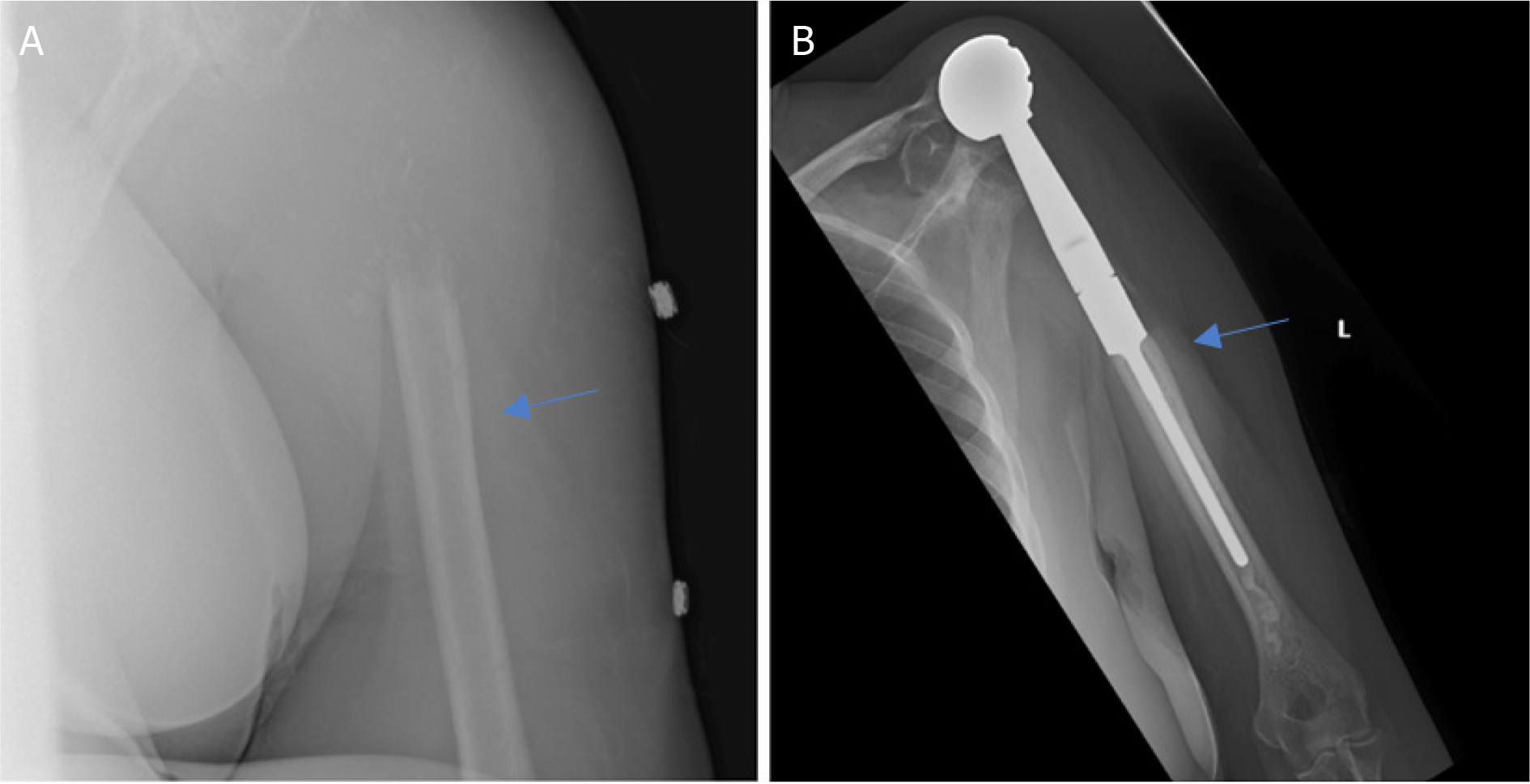
Anteroposterior radiographs of a 61-year-old female with: multiple myeloma of the proximal humerus preoperatively (A); five years after wide excision and proximal endoprosthesis reconstruction (B). Blue arrow notes the junction between the prosthesis and native bone.

Metastatic bone disease and myeloma commonly affect the proximal humerus. Common primary carcinomas that metastasize to bone include prostate, breast, kidney, thyroid, and lung. Breast and lung are the most common in females. Prostate and lung are the most common in males. Myeloma represents the most common primary bone cancer. In the setting of metastatic disease or myeloma involving the proximal humerus, there are limited indications for resection. Indications in these patients include: (1) solitary or oligometastatic renal cell carcinoma [Figures 1 and 2]; (2) extensive proximal humeral bone destruction that makes prophylactic internal fixation unreliable or simply not feasible [Figure 3]; (3) failure of other constructs due to tumor progression; and (4) hardware failure.

### Primary Bone Tumors

The proximal humerus is a common location for primary sarcomas, including osteosarcoma, Ewing sarcoma, and chondrosarcoma^[8]^.

**Figure 4.**
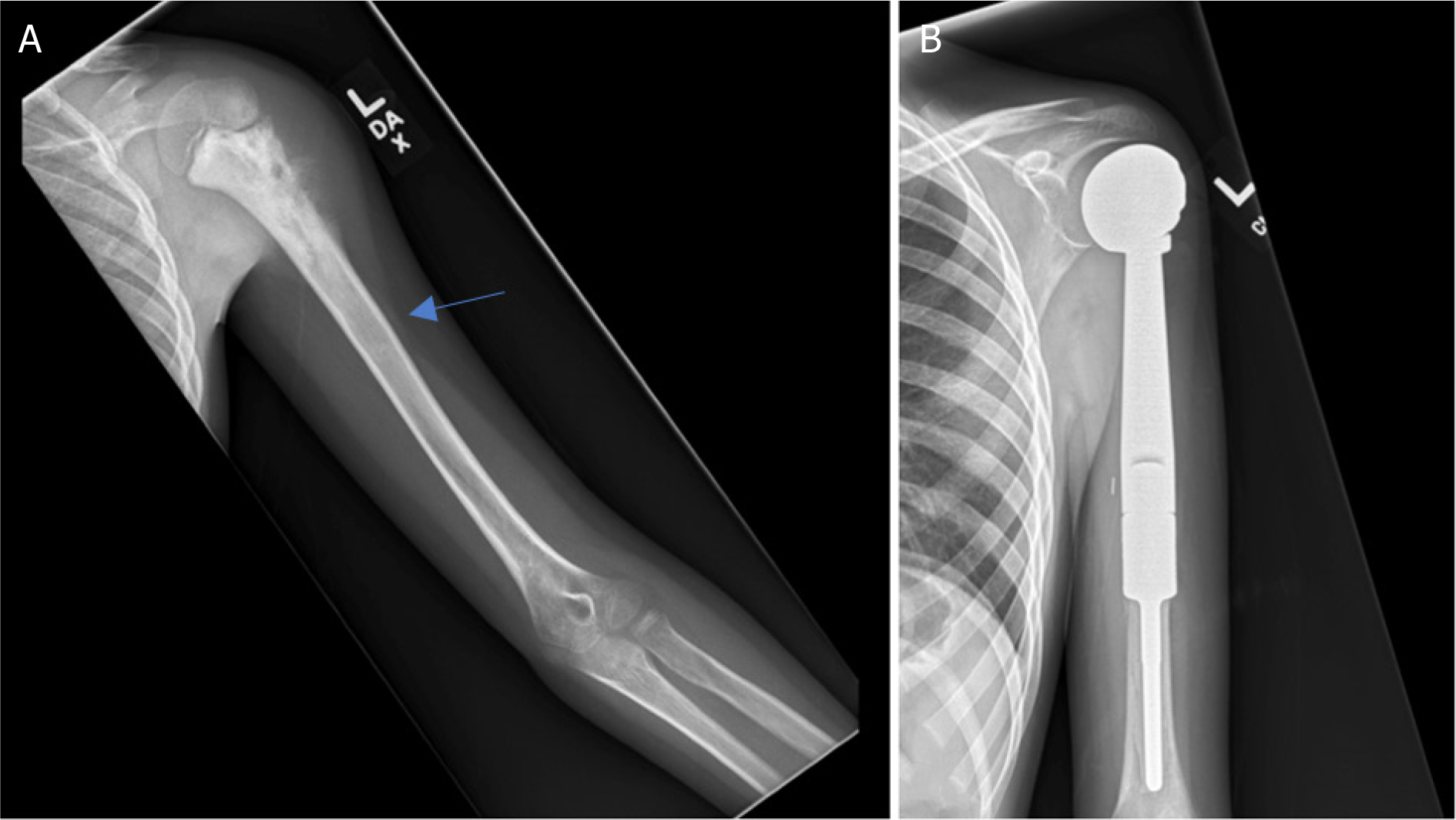
Anteroposterior radiographs of a 10-year-old male with: osteosarcoma of the proximal humerus preoperatively (A); after wide excision and proximal endoprosthesis reconstruction (B) Blue arrow notes the amount of resected bone

Osteosarcoma is the most common bone sarcoma overall and most common in pediatric patients^[8]^. The proximal humerus is the third most common location for osteosarcoma. The standard of care for treatment of osteosarcoma includes wide surgical resection with concomitant chemotherapy [Figure 4]. The typical metaphyseal location lends itself to intra-articular wide resection.

Ewing sarcoma is the second most common pediatric sarcoma^[8]^. Although Ewing sarcoma more commonly involves the diaphysis of long bones, it sometimes involves the metaphysis. The most common locations are the femur and tibia, but it can occur in the humerus. Treatment with surgical resection is an alternative to radiotherapy but favored whenever resected bone can be reconstructed, as it can in the proximal humerus.

**Figure 5.**
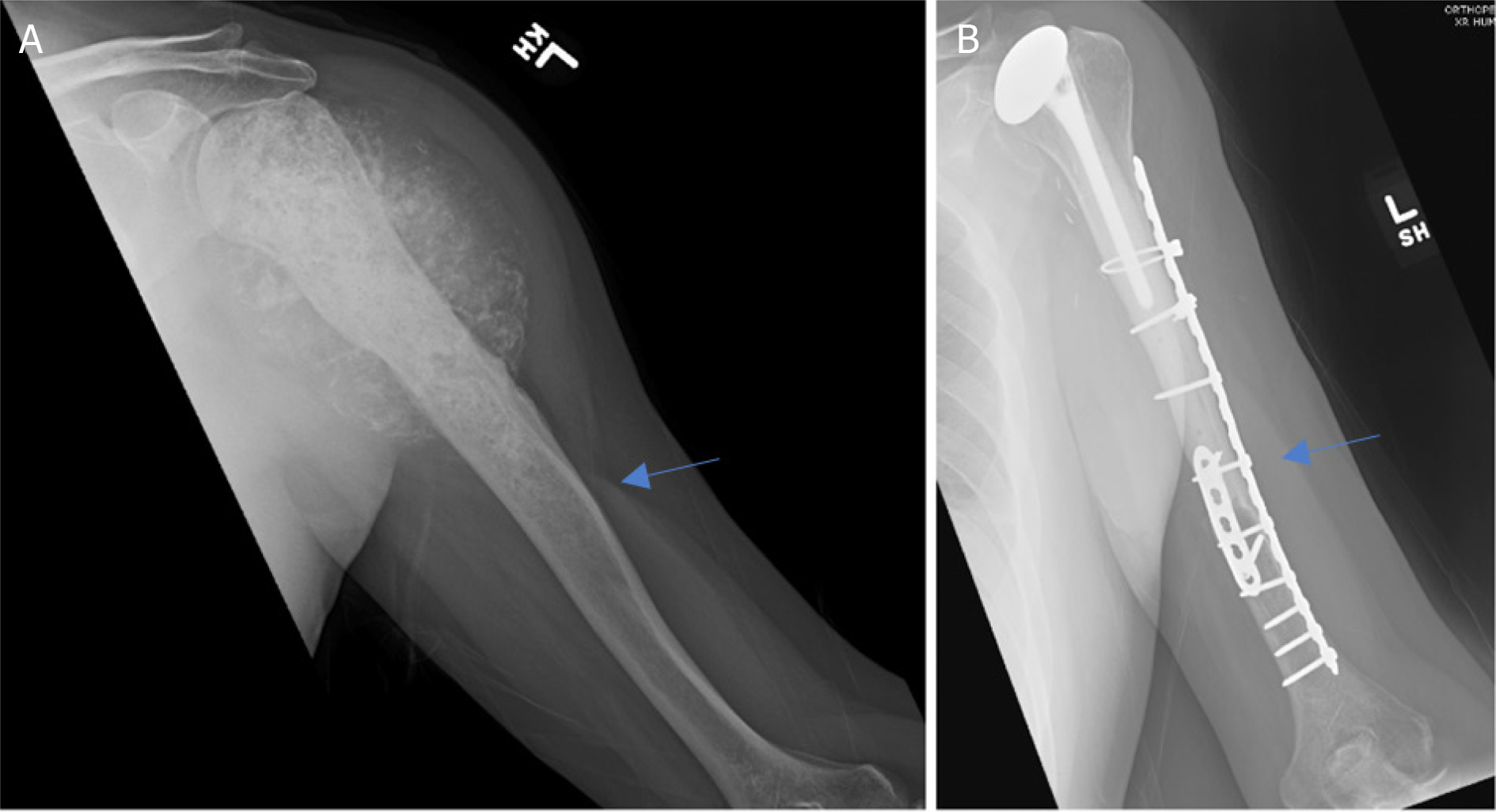
Anteroposterior radiographs of a 57-year-old male with: chondrosarcoma of the proximal humerus preoperatively (A); three years after wide excision and proximal humeral APC reconstruction (B). Blue arrow notes the junction between the allograft and native bone.

**Figure 6.**
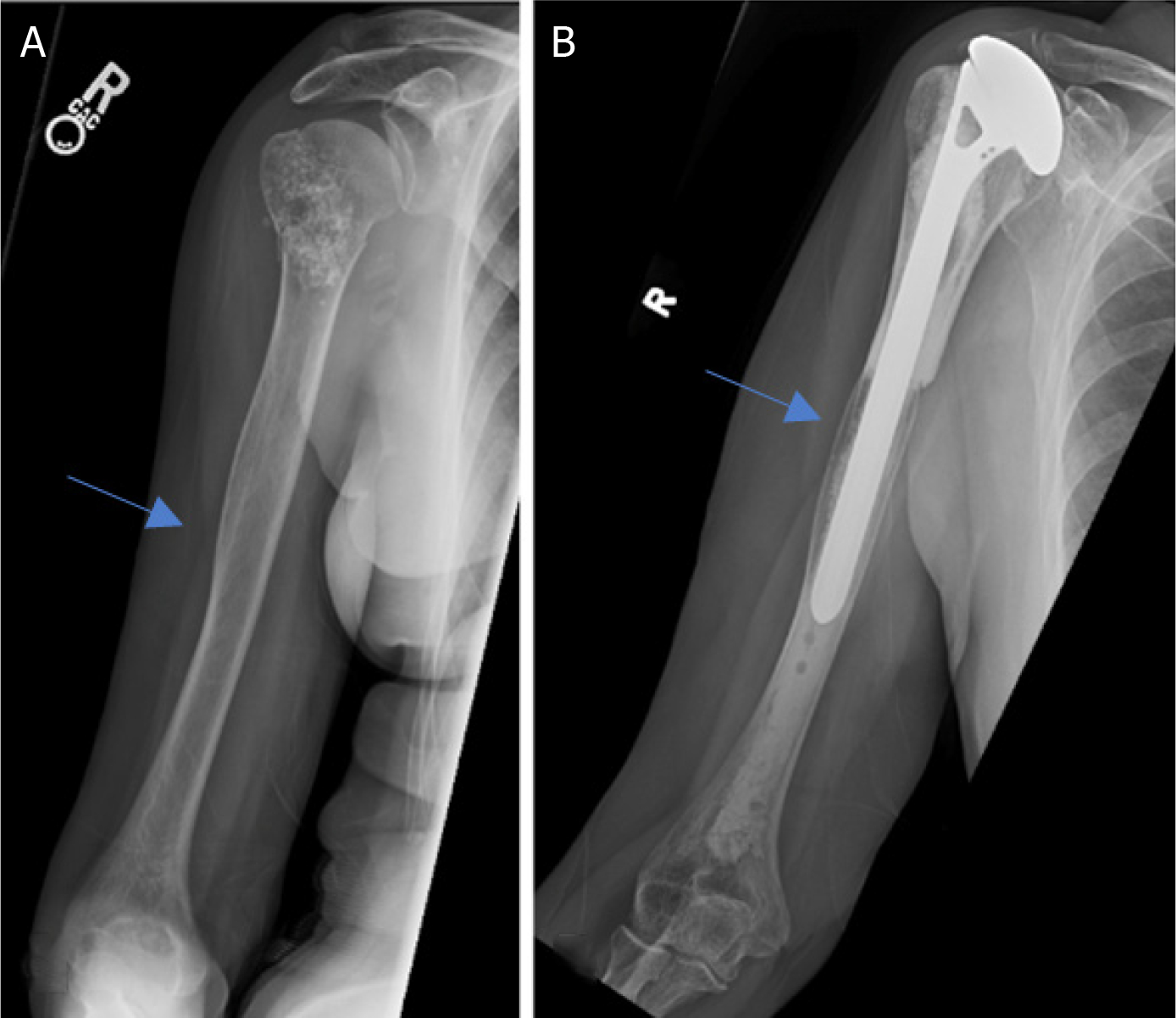
Anteroposterior radiographs of a 65-year-old male with: chondrosarcoma of the proximal humerus preoperatively (A); 4.5 years after wide excision and proximal humeral APC reconstruction (B). Blue arrow notes the junction between the allograft and native bone.

**Figure 7.**
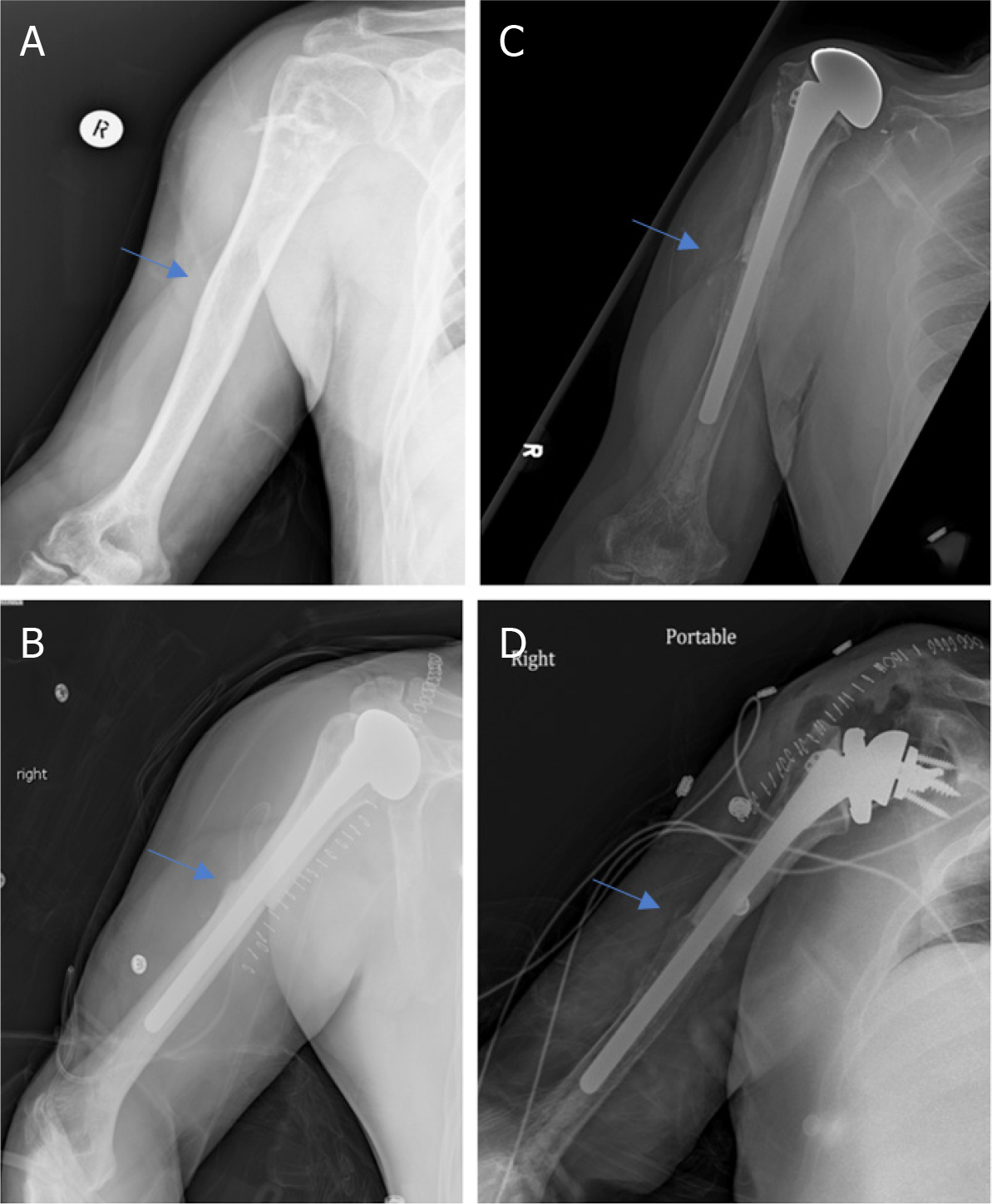
Anteroposterior radiographs of a 57-year-old male with: chondrosarcoma of the proximal humerus preoperatively (A); two months after wide excision and proximal humeral APC reconstruction with hemiarthroplasty (B); Seven years postoperatively, patient represented with excellent integration of the allograft but with proximal migration of the humeral head had worsening anterior superior escape on physical exam (C); anteroposterior radiographs immediately postoperatively after conversion from hemiarthroplasty to RTSA with glenosphere placement and modular exchange of the humeral head component for a humeral tray (D). Blue arrow notes the junction between the allograft and native bone.

Chondrosarcoma is the most common adult bone sarcoma^[8]^. Chondrosarcoma most frequently occurs in the pelvis, proximal femur, and proximal humerus. Treatment of grade 2 and 3 tumors is wide surgical resection [Figures 5–7]. As for osteosarcoma, the typical metaphyseal location lends itself to intra-articular resection.

### Benign locally aggressive tumors

**Figure 8.**
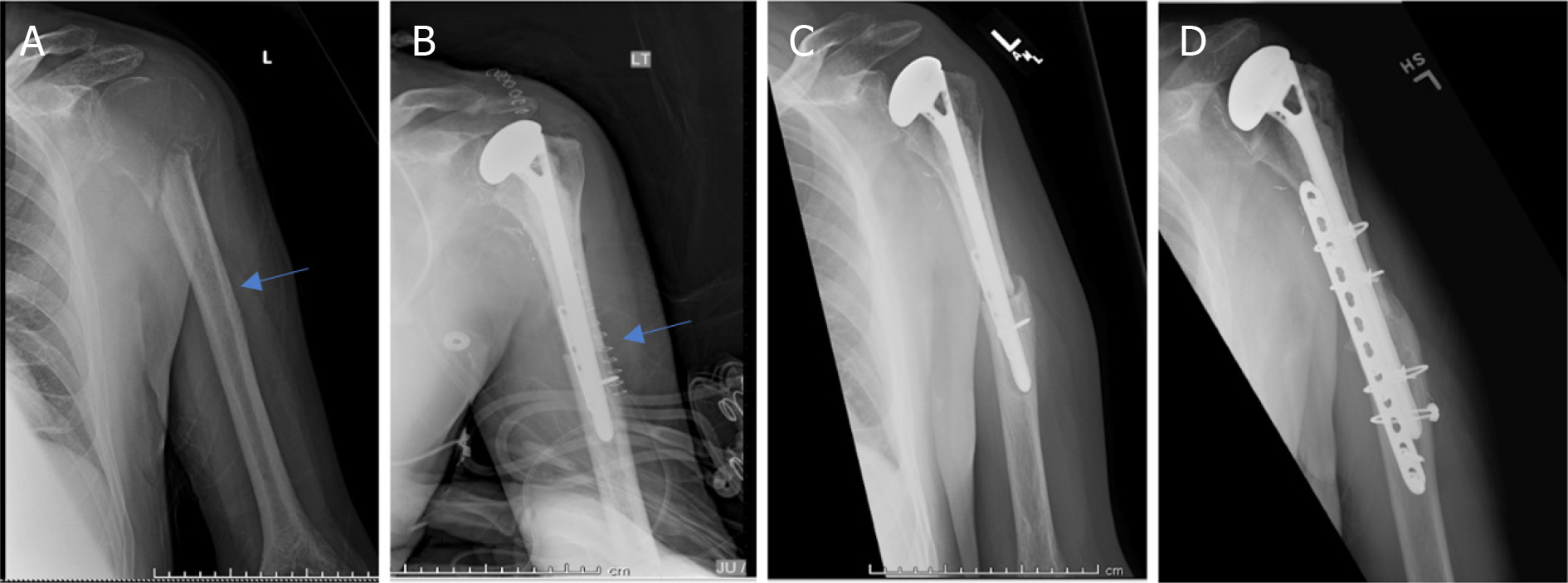
Anteroposterior radiographs of a 55-year-old male with: giant cell tumor of the proximal humerus preoperatively (A); six months after wide excision and proximal humeral APC reconstruction (B); patient was noted to have an allograft-host non-union with hardware failure at the two-year follow-up visit (C); anteroposterior radiographs after bone grafting, repair of the non-union with open reduction and internal fixation (D) Blue arrow notes that junction between allograft and native bone.

Aggressive benign tumors with extensive bone destruction may necessitate resection if in the proximal humerus. While these indications are rare, giant cell tumor of the bone is the most likely benign tumor to require resection [Figure 8]. Rarely, aneurysmal bone cyst, osteoblastoma, and chondromyxoid fibroma require resection. Aneurysmal bone cyst is unlikely to require resection and reconstruction in pediatric patients, but they are particularly aggressive, secondary, or recurrent in an adult patient. Osteoblastoma is rare in the proximal humerus and necessitates resection only when it behaves aggressively, such as when it is hard to differentiate from osteosarcoma. Chondromyxoid fibroma is also an uncommon proximal humeral tumor but has the potential to behave aggressively.

## OPERATIVE OPTIONS

### Osteoarticular allograft

**Figure 9.**
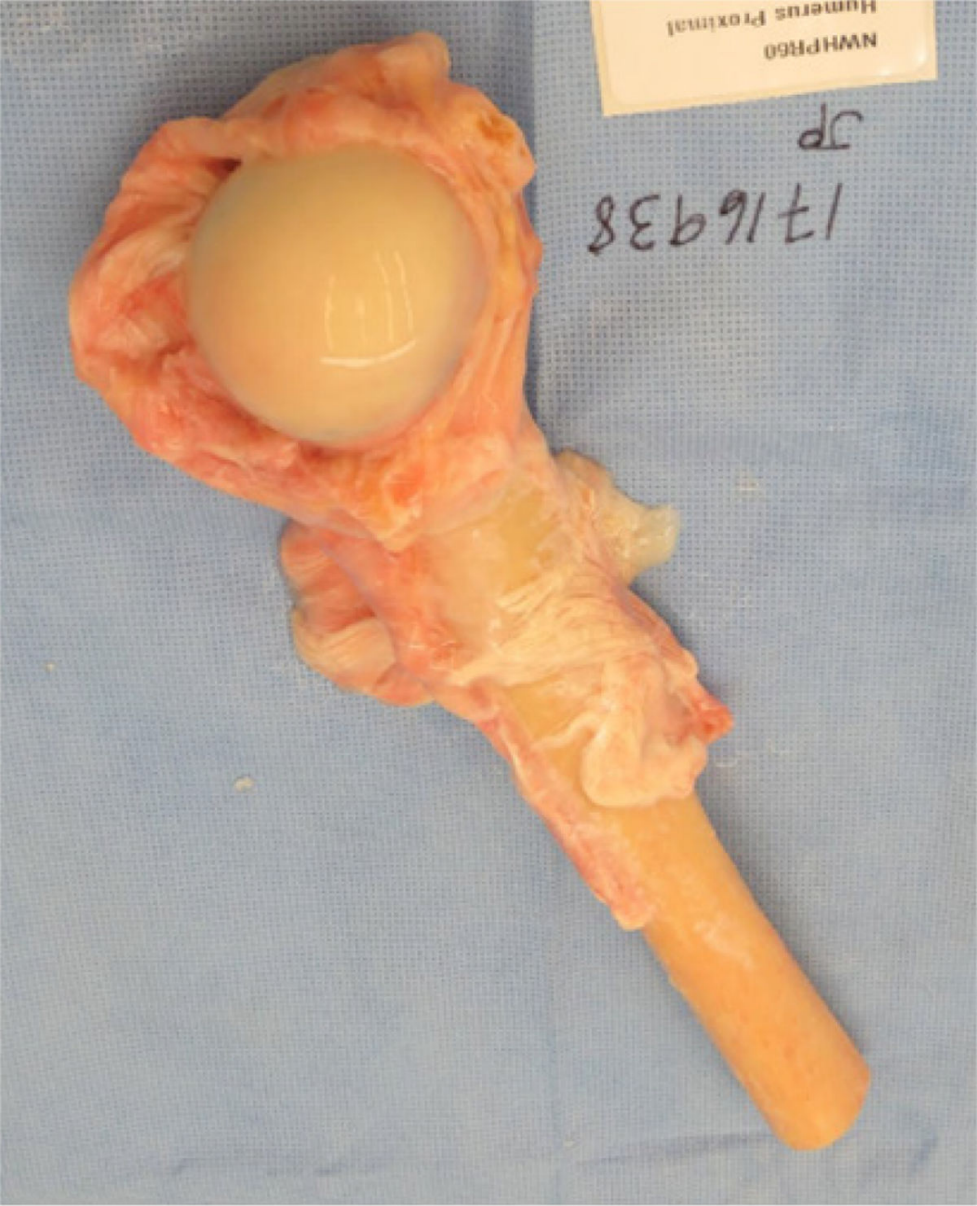
An example of a fresh frozen proximal humeral osteochondral allograft with preserved soft-tissue attachments. This allograft can be used in osteochondral allograft and allograft composite prosthesis reconstruction techniques

Reconstruction of the proximal humerus using an osteoarticular allograft [Figure 9] is a completely biologic articular reconstruction option.

#### Indications:

Osteoarticular allograft reconstruction can be considered in pediatric and young patients due to concerns with prosthesis-related complications^[9]^. Contraindications include evidence of intra-articular tumor, inadequate host tissue to reconstruct the glenohumeral joint, and pre-existing glenohumeral arthrosis. A relative contraindication is need for radiotherapy, which may lead to non-union at the graft-host interface.

#### Technique:

Although tissue matching is not required, size matching is important. The planned resection of the humerus should be measured on advanced imaging. This is important to ensure appropriate length allograft is ordered and available. To preserve mechanical strength, non-irradiated bone should be used. To facilitate soft-tissue reconstruction, an allograft with soft-tissue attachments should be used. During surgery, the allograft can be taken out of the packaging and placed directly in a warm normal saline solution [Figure 9]. During the resection of the tumor, care should be taken to preserve the uninvolved glenohumeral capsule and tendon stumps of all the muscles resected, when possible. The glenohumeral capsule and the respective tendon stumps should be tagged with sutures for later identification. The humeral osteotomy should be completed in a transverse or step-wise fashion. The length of the resected native humerus should be measured.

The allograft should be tailored and cut to fit the native humeral osteotomy. It is important to have adequate length of the allograft humeral height to maintain adequate tension of the retained and repaired soft-tissue structures. The prepared allograft is placed into the proximal humeral defect, aligned, and any modification of the distal cut of the allograft should be performed. Next, the posterior, inferior, and anterior glenohumeral native capsule should be repaired to the allograft capsule with non-absorbable suture. The host rotator cuff can be repaired to the allograft rotator cuff attachments. This should provide enough stability of the glenohumeral joint to reduce the distal end of the allograft bone to the host humeral osteotomy site. Anatomic reduction of the host and donor bone is critical. A lateral locking compression plate is used for fixation. This fixation can be supplemented with an additional anterior shorter plate at 90 degrees to the lateral plate for additional biomechanical strength of the construct. Holes in the allograft should be minimized to avoid increased fracture risk but adequate for rigid fixation. Remaining transected tendons can be attached to the allograft soft-tissue attachments, particularly the latissimus dorsi, teres major, pec major, and deltoid^[9]^. Postoperatively, the patient is placed in a sling. Initially, the patient is non-weightbearing, and range of motion to the shoulder is limited. Once the soft-tissue healing is noted to be adequate, pendulum exercises and active assisted range of motion exercises can be initiated.

#### Advantages [Table 4]:

The advantages of an osteoarticular allograft reconstruction include anatomic restoration of bone stock, glenohumeral joint capsule, rotator cuff, and surrounding dynamic shoulder stabilizers^[10]^. Presence of the soft-tissue attachments on an osteoarticular allograft facilitates host-to-graft soft-tissue repair^[11]^. Over time, host-to-graft healing can occur with the host tissue healing to the soft-tissue bone components of the allograft, unlike with a metallic endoprosthesis.

#### Disadvantages [Table 4]:

Reported complication rates have been relatively high^[9,10]^. Allograft specific complications include allograft osteolysis, non-union, hardware failure, resorption, subchondral collapse, glenohumeral arthrosis, and allograft fracture. These specific complications are related to the use of allograft and the potential problems with using allograft tissue for reconstruction. Ubiquitous potential problems with resection/reconstruction of the proximal humerus include glenohumeral subluxation or dislocation, infection, wound healing problems, and local recurrence. Additionally, development of glenohumeral arthrosis can occur after osteochondral allograft reconstruction and result in continued pain and dysfunction^[12]^. In addition to complications, appropriately sized and procured osteoarticular allografts may be difficult to locate depending on access to tissue banks and available stock. The treatment of these potential complications is case dependent.

#### Outcomes:

A 1990 series of 16 patients from Massachusetts General reported 68% survivorship at 5 years^[13]^. Mean MSTS score decreased from 81% at 14 months to 70% at 34 months^[13]^. In a 2015 series of 19 patients from the Buenos Aires, graft survival rate was 55% at 10 years with mean MSTS score of 76% in patients with retained allografts^[9]^.

### Endoprosthesis

Reconstruction of the proximal humerus may also be done with an endoprosthesis construct using readily available modular implants [Figures 3 and 4]. Their modularity allows for restoration of humeral height, soft-tissue tensioning, and amount of constraint but does not provide the potential for soft-tissue healing to the reconstruction.

#### Indications:

Endoprosthesis reconstructions should be considered in patients who have intra-articular tumor involvement and those with limited expected survival who need immediate stability and early functionality. Most endoprosthesis implants are usually implanted as hemiarthroplasties [Figures 3 and 4], but some modern endoprosthesis implants have the convertibility to be placed in a reverse total shoulder arthroplasty (RTSA) configuration and accommodate a glenosphere.

#### Technique:

As for any planned proximal humeral resection/reconstruction, the planned length of resection should be measured on preoperative advanced imaging, and preservation of soft tissues that can be repaired during the reconstruction should be accomplished during the resection. The deltoid insertion should be spared if possible. The glenohumeral capsule and the respective tendon stumps should be tagged with sutures for later identification. These structures are useful in achieving joint stability by attaching these soft-tissue attachments to the endoprosthesis. After measuring the resected specimen, the defect is reconstructed by combining the modular humeral head, body, and shaft endoprosthetic components to achieve optimal soft-tissue tensioning. If adequate distal fixation is not achieved with uncemented intramedullary stem, cemented techniques should be used.

Soft-tissue repair to endoprosthesis can utilize attachment of soft tissue proximally to holes in the endoprosthesis with non-absorbable sutures. Soft-tissue repair can be augmented by an aortic synthetic mesh graft placed over the endoprosthesis to which the soft tissues can be sutured^[14]^.

In RTSA endoprostheses, adequate lateralization and standard glenosphere placement techniques should be used. Since endoprosthetic reconstruction after oncologic resection has a higher instability risk compared to non-oncology patients, when using a RTSA, techniques to increase stability include increased lateralization of the glenosphere, a larger glenosphere, and a constrained humeral insert.

Postoperatively, the patient is placed in a sling. Initially, the patient is non-weightbearing and range of motion to the shoulder is limited. Often with endoprosthesis reconstruction, immediate light weightbearing and active assisted range of motion exercises can be initiated.

#### Advantages [Table 5]:

The advantages of endoprosthesis reconstruction are modularity, early return to function, lower reported complication rates, and superior implant survival^[15–17]^. This reconstruction option also eliminates allograft specific concerns including allograft-host integration, subchondral collapse, allograft fractures, and non-union.

#### Disadvantages [Table 5]:

The disadvantages of endoprosthesis reconstruction include increased risk for dislocation, proximal migration [Figure 3], and infection. Decreased capacity for restoration of soft-tissue attachments due to the non-biologic metal-soft-tissue interface contributes to the potential for instability^[16,18]^. Proximal migration [Figure 3], subluxations, and dislocations are common with this reconstruction^[19–21]^. Ross *et al.*^[18]^ reported proximal migration, subluxation or dislocations in 16/25 endoprosthetic proximal humeral reconstructions. Endoprosthetic complication rates between 4.5% and 85% have been reported, with the most common complications including proximal migration, subluxation, infection, and aseptic loosening^[17]^.

#### Outcomes:

This reconstruction technique can result in good functional outcomes, as shown in a systemic review performed in 2014^[17]^. In this systemic review, MSTS functional scores ranged from 61% to 77% over 10 studies in 141 patients^[17]^. Implant survival ranged from 38% to 100% over 17 studies in 341 patients^[17]^. In another case series of 39 patients, Raiss reported 72% survivorship with average three-year follow-up^[22]^.

### Allograft Prosthetic Composite

Reconstruction of the proximal humerus using an allograft prosthetic composite (APC) provides a hybrid of allograft and prosthetic reconstruction techniques [Figure 5]. The APC was initially designed in hip and knee surgery to address the problems with resorption, fracture, and cartilage degeneration in osteochondral allograft reconstructions. This reconstruction option has a hybrid advantage and disadvantage profile combining some of the advantages and disadvantages of both the all biologic osteochondral allograft and the endoprosthesis reconstructive options.

#### Indications:

In patients requiring substantial bone and soft-tissue resections, an APC can help decreased the risk of dislocations and instability associated with an endoprosthesis reconstruction. In situations where there is tumor involvement of the glenohumeral joint, an extra-articular resection is needed to limit the risk of local recurrence. If there is pre-existing glenohumeral arthrosis in conjunction with significant soft-tissue resection, an APC can be a viable reconstruction option. In situations where the rotator cuff has to be resected or compromised, but the deltoid function can be preserved, a RTSA can be used [Figures 1 and 2]^[17]^. The RTSA is more constrained, and therefore there is decreased risk of proximal migration and instability. The RTSA accomplishes this by moving the center of rotation more inferior and medial allowing the deltoid to act with a longer lever arm.

#### Technique:

Prior to surgery, the planned resection of the humerus should be measured on the preoperative advanced imaging^[23]^. This is important to ensure that the appropriate length proximal humeral allograft is available during the surgery. During the surgery, the allograft can be taken out of the packaging and placed directly in a warm normal saline solution [Figure 9]. During the resection of the tumor, care should be taken to preserve the uninvolved glenohumeral capsule and tendon stumps of all the muscles resected. The deltoid insertion should be spared if possible. The glenohumeral capsule and the respective tendon stumps should be tagged with sutures for later identification. Once the resection and the humeral osteotomy is completed, the humeral length of the resected native humerus should be measured. The osteotomy should be completed in either a transverse fashion or a step-wise fashion. Next, the humerus allograft should be prepped on the back table. The allograft should be tailored and cut to fit the native humeral osteotomy. It is important to have adequate length of the allograft humeral height to maintain adequate tension of the repaired soft-tissue structures. Next, the allograft should be prepped on the back table using the implant system of choice. The allograft should be prepped according to the appropriate broaches and reamers to accommodate the chosen implant. At this point, if a RTSA is being completed, the glenosphere should be placed. In the case of using a RTSA, adequate lateralization and standard glenosphere placement techniques should be used. Since these patients are at a higher risk for instability when compared to non-oncology patients, techniques to increases stability of the RTSA should be used including increased lateralization of the glenosphere, placement of a larger glenosphere, and using a constraint humeral insert. Next, the implant with the appropriate humeral stem implant should be implanted into the allograft. The implant should be long enough to incorporate approximately two cortical diameters distal to the native humeral osteotomy site. Next, the distal portion of the implant should be placed into the native humeral canal. Cement techniques should be used to fix the distal portion of the implant into the native humeral canal. Prior to final cementation, the humeral length and soft-tissue tensioning should be trailed using a trial prosthesis. Then, the distal portion of the implant is cemented into the native humeral bone. The decision to place a supplementary plate can be made at the discretion of the surgeon [Figure 5]. A locking compression plate can be used for fixation. This fixation can be supplemented with an additional anterior based short plate. Next, the posterior, inferior, and anterior glenohumeral native capsule should be repaired to the allograft capsule with non-absorbable suture. The host rotator cuff should be repaired to the allograft rotator cuff attachments. Next, the remaining resected muscles can be attached including the latissimus dorsi, teres major, pec major, deltoid, etc. Finally, the remaining superficial soft-tissue structures can be closed.

Postoperatively, the patient is placed in a sling. Initially, the patient is non-weightbearing and range of motion to the shoulder is limited. Active assisted shoulder range of motion can be initiated after the first week. Once healing of the allograft-host interface is noted to be adequate, weightbearing can progressively increased.

#### Advantages [Table 6]:

The advantages of this type of reconstruction include anatomic restoration of the bone stock, glenohumeral joint, and surrounding soft tissues. This reconstruction option allows for host-to-graft soft-tissue attachments to attach host tendons, including the latissimus dorsi, deltoid, rotator cuff, etc^[11]^. Over time, the soft-tissue attachments and bony components of the allograft can be fully incorporated into the host bone and soft tissues over time, unlike with a prosthesis. Additionally, this reconstruction method can address any pre-existing glenohumeral arthrosis. Placement of standard hemiarthroplasty or RTSA implants can be used which allows for more modularity and lower cost. The patient also gets immediate stability and early functionality of the shoulder compared to an osteoarticular allograft.

#### Disadvantages [Table 6]:

The disadvantages include allograft-related complications including allograft osteolysis, non-union [Figures 2 and 8], hardware failure, allograft fracture, allograft resorption, stress shielding, and delayed union^[24]^. Additionally, instability, aseptic loosening, and proximal migration [Figures 6 and 7] should be considered^[25]^. Availability of tissue banks and variety of allograft options to include soft-tissue attachments and enough resected bone stock can be variable depending on geographic location.

#### Outcomes:

A case series of 36 patients treated with APC with an average follow-up of six years showed that the average MSTS score at the last follow-up was 26 out of 30 points with an 88% survivorship at 10 years^[25]^. Reconstruction with RTSA has shown good functional outcomes as well, with mean MSTS scores reported from 60% and 79% in different case series^[15,26–28]^. In general, numerus studies have shown better MSTS scores and better abduction in patient with intact deltoid^[24–27]^.

### Other Surgical Options

Other proximal humeral reconstruction options include the Clavicula pro humero and glenohumeral arthrodesis. Alternatively, a Tikhoff-Linberg procedure can be performed for salvage cases. The indications for these procedures are rare.

## CONCLUSION

Limb salvage surgery with wide resection and reconstruction is considered the standard of care for a number of oncologic indications including primary bone tumors and metastatic disease. Wide resections of the proximal humerus and the surrounding soft-tissue stabilizing structures necessitate a complex reconstruction. While there are a number of case series reviewing different types of reconstruction options after proximal humeral resection for tumor, there are no high-level comparative studies in the literature. Considering this, there is little agreement on the ideal method, but reverse total shoulder reconstruction has been increasingly used.

## Figures and Tables

**Table 1.** Malawer surgical classification system of limb-sparing resections of the shoulder girdle

Type I	Intra-articular proximal humeral resection
Type II	Partial scapulectomy
Type III	Intra-articular total scapulectomy
Type IV	Extra-articular scapular and humeral head resection
Type V	Extra-articular humeral and glenoid resection
Type VI	Extra-articular humeral and total scapular resection

**Table 2.** Musculoskeletal tumor society functional evaluation (upper limb)

Score	Pain	Function	Emotional acceptance	Hand positioning	Manual dexterity	Lifting ability

5	No pain	No restriction	Enthused	Unlimited	No limitations	Normal load
4	(Intermediate)	(Intermediate)	(Intermediate)	(Intermediate)	(Intermediate)	(Intermediate)
3	Modest/non-disabling	Recreational restriction	Satisfied	Not above shoulder	Loss of fine movements	Limited (minor load)
2	(Intermediate)	(Intermediate)	(Intermediate)	(Intermediate)	(Intermediate)	(Intermediate)
1	Moderate/intermittently disabling	Partial occupational restriction	Accepts	Not above waist	Cannot pinch	Helping only(cannot overcome gravity)
0	Severe/continuously disabling	Total occupational restriction	Dislikes	None	Cannot grasp	Cannot move

**Table 3.** Henderson *et al.*^[5]^ classification of complications

Type of failure	Definition

1	Soft tissue failure
2	Aseptic loosening
3	Structural failure
4	Infection
5	Tumor progression

**Table 4.** Advantages and disadvantages of osteochondral allograft reconstruction

ADVANTAGES	DISADVANTAGES

All-biologic reconstruction	Allograft osteolysis
Host-graft soft tissue attachments	Non-union
Continued boney and soft-tissue integration	Hardware failure
	Allograft fracture
	Allograft resorption
	Subchondral collapse
	Glenohumeral arthrosis
	Availability of allograft
	Poor long-term survivability

**Table 5.** Advantages and disadvantages of endoprosthesis reconstruction

Advantages	Disadvantages

Modularity of prosthesis	Proximal Migration (Hemiarthroplasty)
Convertibility of prosthesis	Instability
Immediate stability	Aseptic Loosening
Early range of motion and functionality	Metal-soft tissue attachments
Lower complication rates compared to osteochondral allograft	Infection
No need for allograft	
Good long-term survivability	
Good long-term functional outcomes	

**Table 6.** Advantages and disadvantages of allograft prosthetic composite reconstruction

Advantages	Disadvantages

All-biologic reconstruction	Allograft osteolysis
Host-Graft soft tissue attachments	Non-union
Modularity of prosthesis	Hardware failure
Convertibility of prosthesis	Allograft fracture
Immediate stability	Allograft resorption
Early range of motion and functionality	Proximal migration (Hemiarthroplasty)
Lower complication rates compared to osteochondral allograft	Instability
Can use standard implants instead of endoprosthesis implants	Aseptic loosening
Good long-term survivability	Availability of allograft
Good long-term functional outcomes	
